# lncRNA MEG3 aggravated neuropathic pain and astrocyte overaction through mediating miR-130a-5p/CXCL12/CXCR4 axis

**DOI:** 10.18632/aging.203592

**Published:** 2021-10-05

**Authors:** Jiacai Dong, Rui Xia, Zhonggui Zhang, Cheng Xu

**Affiliations:** 1 Department of Anesthesiology, Qianjiang Hospital Affiliated to Renmin Hospital of Wuhan University, Qianjiang 433100, Hubei, China; 2 Department of Anesthesiology, The First People's Hospital of Jingzhou, Jingzhou 434000, Hubei, China; 3 Department of Pain, The First People's Hospital of Jingzhou, Jingzhou 434000, Hubei, China

**Keywords:** MEG3, miR-130a-5p, neuropathic pain, CXCL12, astrocytes

## Abstract

Objective: Long non-coding RNAs (lncRNAs) exert a critical function in mediating neuropathic pain (NP). MEG3, a novel lncRNA, contributes to astrocyte activation and inflammation. However, its role in NP remains unclear.

Methods: The chronic constriction injury (CCI) method was employed to construct an NP rat model. Astrocyte activation was induced by lipopolysaccharide (LPS). The profiles of MEG3, microRNA (miR)-130a-5p, CXC motif chemokine receptor 12 (CXCL12)/CXC motif chemokine receptor 4 (CXCR4), and the Rac1/NF-κB pathway in CCI rats’ spinal cord tissues and astrocytes were monitored by reverse transcription-quantitative PCR (RT-qPCR) and western blot (WB). Pain scores of CCI rats were assessed. Enzyme-linked immunosorbent assay (ELISA) was adopted to monitor neuroinflammation alteration. The glial fibrillary acidic protein (GFAP)-labeled astrocytes were tested by immunohistochemistry (IHC). Bioinformatics, dual-luciferase reporter assay and RNA immunoprecipitation (RIP) were utilized to verify the molecular mechanism between MEG3 and miR-130a-3p.

Results: MEG3, CXCL12 and CXCR4 were overexpressed and miR-130a-5p was knocked down in CCI rats and LPS-induced astrocytes. Up-regulating MEG3 aggravated NP, enhanced inflammatory cytokines interleukin-1β (IL-1β), tumor necrosis factor (TNF)-α, and interleukin-6 (IL-6) expression and release in CCI rats and LPS-induced astrocytes. Up-regulating miR-130-5p repressed LPS-induced inflammation in astrocytes. AS verified by the dual-luciferase reporter assay and RIP assay, MEG3 sponged miR-130a-5p as a competitive endogenous RNA (ceRNA). What’s more, miR-130a-5p up-regulation weakened the MEG3-induced proinflammatory effects on LPS-induced astrocytes.

Conclusions: MEG3 aggravates NP and astrocyte activation via the miR-130a-5p/CXCL12/CXCR4 axis, which is a potential therapeutic target for NP.

## INTRODUCTION

Neuropathic pain (NP) is a chronic secondary pain resulting from illness or injury to the nervous system, affecting up to 8% of the population [1]. NP is commonly caused by diabetes, reactivation of herpes zoster, nerve compression or radiculopathy, alcohol consumption, chemotherapy or certain drug abuse, and trigeminal neuralgia [2]. It is characterized by an abnormal hypersensitivity to stimuli (hyperalgesia) and a nociceptive response to non-harmful stimuli (abnormal pain) [3]. Neuroinflammation is closely related to NP, and inflammation-induced microglial activation and astrocytes contribute to NP evolvement [4]. Current recommended first-line therapies include antidepressants (tricyclic agents and serotonin-norepinephrine reuptake inhibitors) and anticonvulsants (gabapentin and pregabalin) [5]. However, in some cases, medication alone cannot control chronic pain. Therefore, it is of great significance to find new treatments for NP.

Long non-coding RNAs (lncRNAs) are small RNAs, which are reported to be closely linked to NP [6]. For example, lncRNA SNHG1 is up-regulated in rats undergoing spinal nerve ligation (SNL), and inhibiting SNHG1 alleviates NP [7]. LncRNA GAS5 is down-regulated in chronic constriction injury (CCI) rats, and GAS5 overexpression represses the expression of inflammatory factors in spinal cord tissues and alleviates NP [8]. Also, lncRNA MRAK009713 [9], KCNA2-AS [10], LncRNA H19 [11], etc., participate in the evolvement of NP. As a lncRNA, maternal expression gene 3 (MEG3) functions a role in mediating inflammation [12]. On the one hand, up-regulating MEG3 hampers inflammatory factor production and improves several diseases, such as rheumatoid arthritis [13], ankylosing spondylitis [14] and osteoarthritis [15]. In parallel, MEG3 increases the expression of inflammatory factors in some diseases, including neurological diseases. For instance, enhanced MEG3 level was detectable in mice subjected to cerebral ischemia-reperfusion (I/R) injury and BV2 cells under oxygen-glucose deprivation (OGD)/R treatment. Forced MEG3 overexpression promotes “M1” microglia polarization and inflammatory factor release [16]. In the neonatal mice suffered from hypoxic-ischemic brain damage (HIBD, MEG3 gained a boosted level. MEG3 knockdown declined the neuronal apoptosis rate and the degree of brain atrophy in HIBD mice, enhanced the learning and memory of neonatal HIBD mice [17]. Similarly, MEG3 is up-regulated in the brain tissue of intracerebral hemorrhage (ICH) rats. Abating MEG3 expression restrains edema, neuronal apoptosis, and the release of inflammatory cytokines and oxidative stress in the brain tissue of ICH rats [18]. Therefore, MEG3 can play a role in neuroinflammation.

C-X-C motif chemokine ligand 12 (CXCL12), also known as stromal cell-derived factor-1 (SDF-1), is a CXC chemokine subfamily member and is widely expressed in diversified tissues and cells. CXCL12 specifically interacts with ligands for the G-protein coupled receptors, including CXC motif chemokine receptor 4 (CXCR4) and CXCR7 [19]. Previous studies have exhibited that the CXCL12/CXCR4 signal is involved in modulating NP. For example, in the SNL-induced rat NP model, CXCL12 and CXCR4 are up-regulated in astrocytes and spinal dorsal horn neurons. Inhibiting CXCL12 relieves NP of the SNL rat model [20]. Huang w et al. found that CXCR4 was up-regulated in L4/5 dorsal root ganglion (DRG) in HIV gp120 protein-mediated NP, and inhibiting CXCR4 expression impeded NP [21]. The above studies conclude that the CXCL12/CXCR4 expression is heightened in the NP model, and intervention of CXCL12/CXCR4 may be an effective method in treating NP.

It is well known that lncRNAs sponge miRNAs, thus declining miRNAs’ regulatory effects on mRNAs. As the report goes, MEG3 suppresses inflammatory progression by regulating miRNA expression [22]. Additionally, miR-130a-5p is closely related to the inflammatory response [23], and MEG8 reduces the neurological impairment of middle cerebral artery occlusion (MCAO) rats by targeting miR-130a-5p [24]. However, the relationship between MEG3 and miR-130a-5p remains elusive. On the other hand, our study revealed that miR-130a-5p relieves NP following SCI via targeting CXCL12 [25]. Hence, we suppose that MEG3 modulates the CXCL12 expression by targeting miR-130a-5p to dampen CCI-induced NP and neuroinflammation.

## MATERIALS AND METHODS

### Animals

Forty adult SD rats (female, four-week-old, 180-200 g) were purchased from Wuhan Animal Experimental Center and randomized into the sham and CCI groups. Rats were raised in standard plastic cages at 24±1° C and humidity of 50-70%, with 12-hour light and dark cycles. They could drink and eat freely. The behavioral experiments were conducted between 10 a.m. and 3 p.m., following the National Institutes of Health Guidelines for the Care and Use of Laboratory Animals to alleviate the rats’ discomfort or stress. All animal tests were authorized by the Animal Research Committee of Jingzhou First People's Hospital.

### The NP rat model

As previously stated [26], the rats were anesthetized with pentobarbital sodium (40 mg/kg), and bilateral sciatic nerve CCI was performed under aseptic conditions. A median thigh incision was made to expose the bilateral sciatic nerve. In contrast, the sciatic nerves in the sham group were exposed and isolated but not ligated. L4 to L6 dorsal spinal cord tissues were harvested on days 0, 1, 3, 5, 7 and 14, respectively.

### Construction of lentiviruses

LV-NC (Catalog No. D03003) and the lentiviral vector of LV-MEG3 were synthesized by Shanghai GenePharma Co., Ltd (Shanghai, China). These lentiviral vectors (1×10^7^/0.05 mL) were then injected into the rats’ tail vein three days before the modeling using a micro-needle [27].

### Pain thresholds assessment

Mechanical pain was evaluated by Von Frey filaments by adopting a paw withdrawal threshold (PWT) [28]. Briefly, rats were put in a clear plastic box with a metal mesh floor. On days 0, 1, 3, 5, 7, and 14 after CCI, calibrated Von Frey filaments (IITC, Woodland Hills, CA, United States) were employed to generate pressure on the plantar surface of the rat's hind paws. Then, the filament size during claw retraction was monitored. The paw withdraw latency (PWL) was adopted to evaluate hyperalgesia on 0, 1, 3, 5, 7, and 14 days after CCI using a foot-pressure measurement instrument. The hind paws were recorded alternately at 5-minute intervals. The duration between stimulation and paw withdrawal was recorded, and the cutoff time was 30 s.

### Immunohistochemistry (IHC)

On the 7th day after the establishment of the CCI rat model, five rats were randomly chosen from each group and executed under chloral hydrate anesthesia. L4 to L6 dorsal spinal cord tissues were collected and stored in 100g/L paraformaldehyde solution and immobilized at room temperature (RT) for 24 hours. Coronal sections (3~4 μM thick) of tissues were placed in an oven for 4~6 hours. Then, the sections were cleaned with PBS three times (2 min each time) and maintained with the rabbit anti-GFAP antibody (Abcam, ab7260, 1: 500, MA, USA) in a refrigerator overnight at 4° C. After washes, the horseradish peroxidase-labeled goat anti-rabbit IgG (1:800) was added and incubated at 37° C for two hours. Afterward, the sections were cleaned, and DAB was adopted for color development. The percentage of area occupied by GFAP-positive astrocytes in the L4 to L6 dorsal spinal cord tissue was assessed with the Image-Pro Plus image analysis software system (Media Cybernetics, MD, USA).

### TdT-mediated dUTP nick end labeling (TUNEL) staining

Paraffin sections were processed following the TUNEL Apoptosis Detection Kit instructions. Three sections were taken from each specimen, and five non-overlapping high magnification views of the L4 to L6 dorsal spinal cord tissue were randomly chosen for each section. The TUNEL-positive cell number, i.e., apoptotic cell number, and the total cell number were counted by adopting the Image-Pro Plus image analysis software. The apoptosis index (AI)=apoptotic cell number/total cell number×100%.

### Cell culture

Normal human astrocytes (NHAs, isolated from the spinal cord) were commercially provided by American Type Culture Collection (ATCC, Rockville, MD, USA) and grown in the astrocyte medium containing 10% fetal bovine serum (FBS, HyClone, Logan, UT, USA) (AM, Cat. #1801, ScienCell). The medium was altered every 2~3 days, and the cells were sub-cultured every 4~5 days.

### Cell transfection

Cells in the logarithmic growth phase were seeded into 6-well plates (5×10^6^cells/well) after trypsinization and sub-culture. Cell transfection was carried out after stable cell growth. The pcDNA empty vector (NC), pcDNA-MEG3 (MEG3), miR-130a-5p mimics and the corresponding counterpart fragments were transfected into astrocytes as per the FuGENE®HD Transfection Reagent (Roche, Shanghai, China) guidelines. Cells were maintained at 37° C with 5% CO_2_. After transfection for 24 hours, cells were exposed to 100 ng/mL LPS for six hours to 14 days. The control group was left untreated.

### Real-time quantitative polymerase chain reaction (qRT-PCR)

MEG3 and miR-130a-5p levels were determined by qRT-PCR. Total RNA was separated using the TRIzol reagent (Invitrogen, CA, USA) and reversely transcribed into first-strand complementary DNA (cDNA) by utilizing the reverse transcription kit (Thermo Fisher Scientific, MA, USA). The miRNA first-strand cDNA synthesis package (Sangon Biotech, Shanghai, China) was adopted for the reverse transcription for miR-130a-3p. The SYBR-Green PCR Master Mix Kit (Applied Biosystems, CA, USA) was employed to amplify the target genes, and the aqMan miRNA analysis kit (Applied Biosystems) was adopted to amplify miR-130a-5p. All amplifications were performed on the 7900HT Fast Real-Time System (Applied Biological System). GAPDH served as the housekeeping gene of MEG3, and U6 served as that of miR-130a-3p. The 2^−ΔΔ-Ct^ method was applied for relative expression analysis. Primer sequences of miR-130a-5p were as follows: forward 5'-AACACGCGCTGACTCCTAGT-3', reverse 5'-CAGTGCAGGGTCCGAGGT-3'. MEG3: forward 5'-GTGAAGGTCGGAGTGAACG-3', reverse 5'- CTCGCTCCTGGAAGATGGTG-3'. GAPDH: forward 5'-CGCTGAGTACGTCGTGGAGTC-3', reverse 5'- GCTGATGATCTTGAGGCTGTTGTC-3'; U6 forward: 5'-GACAGATTCGGTCTGTGGCAC-3', reverse: 5'-GATTACCCGTCGGCCATCGATC-3'.

### Western blot (WB)

After tissue and cell treatment, RIPA lysis buffer (Beyotime Biotechnology, Shanghai, China) was added, and the total protein was isolated. Then, 50 g of total protein was subjected to 12% polyacrylamide gel and electrophoresed at 100 V for two hours. It was then electrically transferred to polyvinylidene fluoride (PVDF) membranes (Millipore, MA, USA). After the membranes were sealed with 5% skimmed milk powder at RT for one hour, they were rinsed with TBST three times (10 min each time) and incubated with primary antibodies (1: 1000, Abcam, MA, USA) of iNOS (ab178945), TLR4 (ab13867), COX2 (ab179800), NF-κB (ab32536), p-NF-κB (ab76302), CXCL12 (ab155090), CXCR4 (ab181020), Rac1 (ab155938), and GAPDH (ab9485) overnight at 4° C. After being rinsed in TBST, the membranes were maintained with the horseradish peroxidase (HRP)-labeled anti-rabbit secondary antibody (concentration 1:3000) for one hour at RT. Subsequently, the membranes were subjected to three washes with TBST (10 min each). At last, WB reagents (Invitrogen) were utilized for color development and imaging, and each protein’s gray intensity was determined by Image J.

### Enzyme-linked immunosorbent assay (ELISA)

L4~L6 dorsal spinal cord tissues were weighed, homogenized by lysate containing protease inhibitors, and centrifuged at 14,000 rpm for 25 min at 4° C to harvest the supernatant. The astrocyte supernatant was collected, centrifuged to remove cell debris, and stored in aliquots at -80° C until being assayed. The interleukin-1β (IL-1β), interleukin-6 (IL-6) and tumor necrosis factor-α (TNF-α) contents in spinal cord tissues and astrocytes were determined by IL-1β, IL-6 and TNF-α ELISA kits (Invitrogen), respectively.

### Data analysis

All experiments were repeated three times. The SPSS17.0 statistical software was employed for statistical analysis of the results. Measurement data were presented as "mean ± standard deviation" (x±s). Student t-test was employed to compare the data between two groups. The one-way analysis of variance (ANOVA) was applied to compare the data among multiple groups, followed by the Tukey post hoc test. *P*< 0.05 indicated statistical significance.

### Ethics statement

Our study was approved by the Animal Research Committee of Jingzhou First People's Hospital.

### Data availability statement

The data sets used and analyzed during the current study are available from the corresponding author on reasonable request.

## RESULTS

### MEG3 was up-regulated and miR-130a-5p was down-regulated in CCI rat models

**Figure 1:**
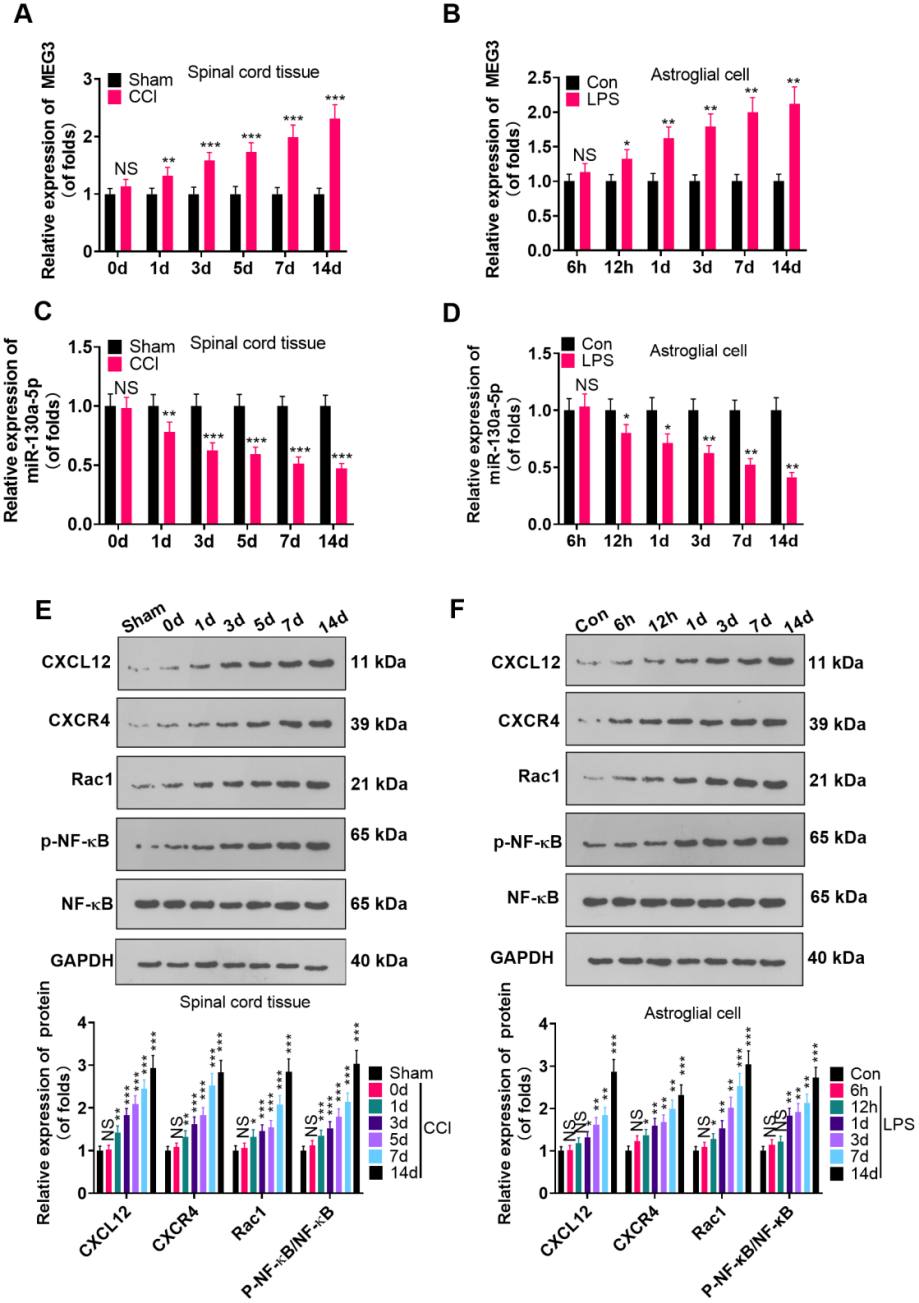
**MEG3 was overexpressed and miR-130a-5p was knocked down in CCI rats.** (**A**, **B**) The expression of MEG3 in the L4-L6 dorsal spinal cord of CCI rats and astrocytes was assayed by RT-qPCR 0, 1, 3, 5, 7, and 14 days after CCI and 6 hours, 12 hours, 1 day, 3 days, 7 days, and 14 days after LPS induction of astrocytes, respectively. (**C**, **D**) Expression of miR-130a-5p in CCI rats and astrocytes was measured by RT-qPCR at 0, 1, 3, 5, 7, and 14 days after CCI and 6 hours, 12 hours, 1 day, 3 days, 7 days, and 14 days after LPS induction of astrocytes, respectively. (**E**, **F**) Expression of CXCL12/CXCR4 and its downstream Rac1 and NF-κB in the dorsal spinal cord of CCI rats and astrocytes at 0, 1, 3, 5, 7, and 14 days and LPS-induced astrocytes at 6 hours, 12 hours, 1 day, 3 days, 7 days, and 14 days, respectively, was measured using WB. Data were expressed as mean±SD. n=5. NS*P*>0.05, *P<0.05, **P<0.01, ***P<0.001 (vs. sham group).

To probe the function of MEG3 and miR-130a-5p in NP, we gauged the expression of MEG3, miR-130a-5p and CXCL12/CXCR4 and its downstream Rac1 and NF-κB in L4-L6 dorsal spinal cord tissues of CCI rats and astrocytes following CCI (0, 1, 3, 5, 7, and 14 days) and LPS induction (6 hours, 12 hours, 1 day, 3 days, 7 days, 14 days), using RT-qPCR and WB. As a result, by contrast with the sham group, the MEG3 expression in CCI rats and astrocytes significantly increased (Figure 1A, 1B; *P*<0.05), the miR-130a-5p expression decreased (Figure 1C, 1D; *P* <0.05), the profiles of CXCL12, CXCR4 and Rac1 were elevated, and the NF-κB phosphorylation was facilitated time-dependently (Figure 1E, 1F; *P*<0.05). These findings demonstrated that MEG3, miR-130a-5p and the CXCL12/CXCR4 pathways were involved in NP.

### Up-regulating MEG3 promoted NP and inflammation

**Figure 2:**
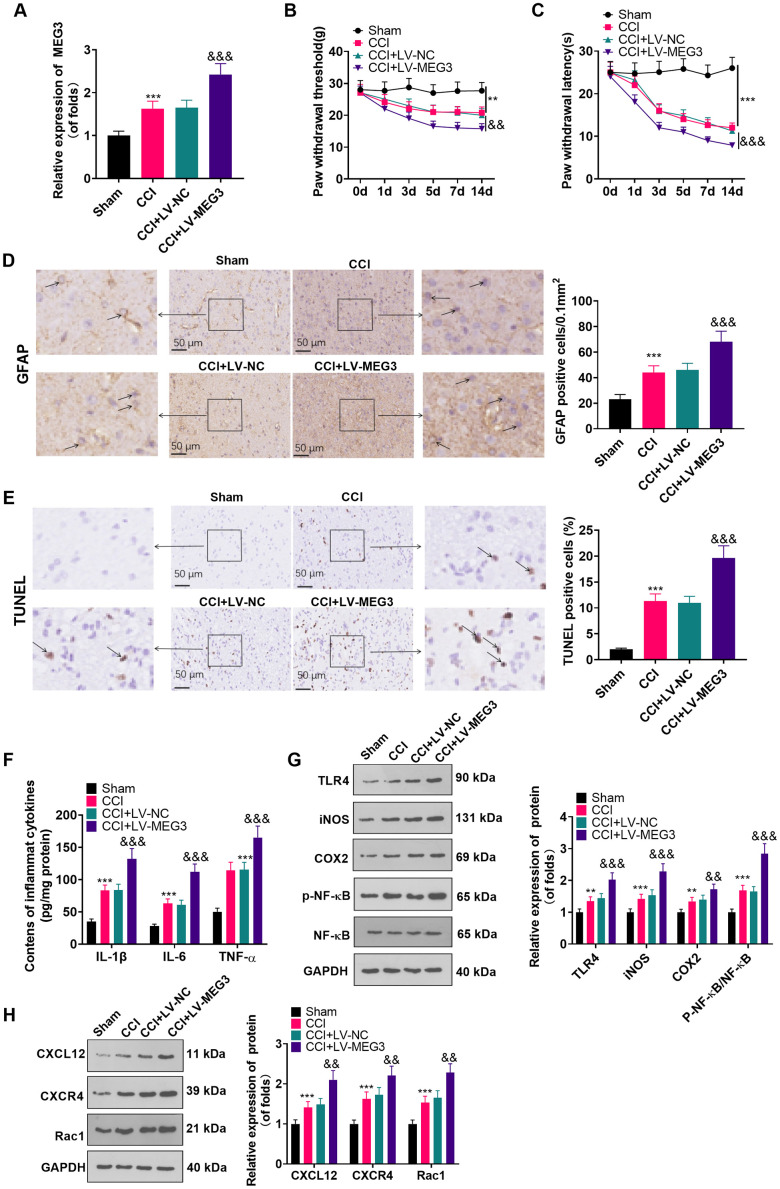
**Up-regulation of MEG3 promoted NP and inflammatory response.** (**A**) After LV-MEG3 transfection, the level of MEG3 in CCI rats was examined by RT-qPCR. (**B**) PWT was adopted to assess the impact of MEG3 on mechanical hyperalgesia. (**C**) PWL was utilized to evaluate the influence of MEG3 on thermal hyperalgesia. (**D**) The proportion of GFAP-positive cells in CCI rats was determined by IHC; Scale bar=50 μm. (**E**) TUNEL staining was applied to detect the influence of MEG3 on neuronal cell apoptosis in the L4-L6 dorsal spinal cord of CCI rats; Scale bar=50 μm. (**F**) Levels of TNF-α, IL-6 and IL-1β in CCI rats after up-regulating MEG3 were examined by ELISA. (**G**, **H**) The contents of TLR4, COX2, iNOS, NF-κB, CXCL12, CXCR4, and Rac1 in dorsal spinal cord tissues of CCI rats after up-regulating MEG3 were monitored by WB. Data were expressed as mean±SD. n=5. ***P<0.001 (vs. Sham group). &*P*<0.05, &&*P*<0.01, &&&*P*<0.001 (vs. CCI+LV-NC group).

We injected recombinant lentivirus LV-MEG3 and LV-NC intrathecally into CCI rats to further probe the function of MEG3 in CCI rats. The MEG3 level was examined by RT-qPCR, which disclosed that the MEG3 expression was elevated after transfection with LV-MEG3 (vs. the CCI+LV-NC group) (Figure 2A; *P*<0.05). The rats’ NP in each group was assessed using PWT and PWL. The outcomes illustrated that the PWT and PWL of CCI rats were declined with time (vs. the sham group). Transfection with LV-MEG3 further boosted mechanical hypersensitivity and thermal hyperalgesia, as evidenced by a significant decrease in PWT and PWL over time (Figure 2B, 2C; *P*< 0.05). Besides, we examined the number of GFAP-responsive astrocytes in the L4-L6 dorsal spinal cord tissue of CCI rats with IHC. It turned out that the proportion of GFAP-responsive astrocytes was heightened in CCI rats, and it was further elevated after transfection with LV-MEG3 (Figure 2D; *P*<0.05). TUNEL was implemented to verify neuronal apoptosis in the L4-L6 dorsal spinal cord tissue of CCI rats, and the results exhibited that the TUNEL-positive cell number was elevated (vs. the sham group) and was further facilitated after the transfection of LV-MEG3 (Figure 2E; *P*<0.05). To figure out whether MEG3 contributed to inflammation in CCI model rats, we tested the expression of inflammatory cytokines and inflammatory proteins in the L4-L6 dorsal spinal cord tissues of CCI rats using ELISA and WB. As a result, by contrast with the sham group, the levels of IL-1β, TNF-α and IL-6 were heightened (Figure 2F; *P* < 0.05), the profiles of TLR4, iNOS, COX2, CXCL12, CXCR4, and Rac1 were elevated, and the NF-κB phosphorylation was increased (Figure 2G, 2H; *P*< 0.05) in the CCI group. What’s more, these inflammatory factors and inflammatory proteins were further augmented in the MEG3 up-regulation model (Figure 2G, 2H; *P*< 0.05 vs. CCI+Lv-NC group). These data indicated that the up-regulation of MEG3 intensified NP and boosted the levels of inflammatory cytokines in CCI rats.

### Up-regulating MEG3 heightened the inflammation of LPS-induced astrocytes

**Figure 3:**
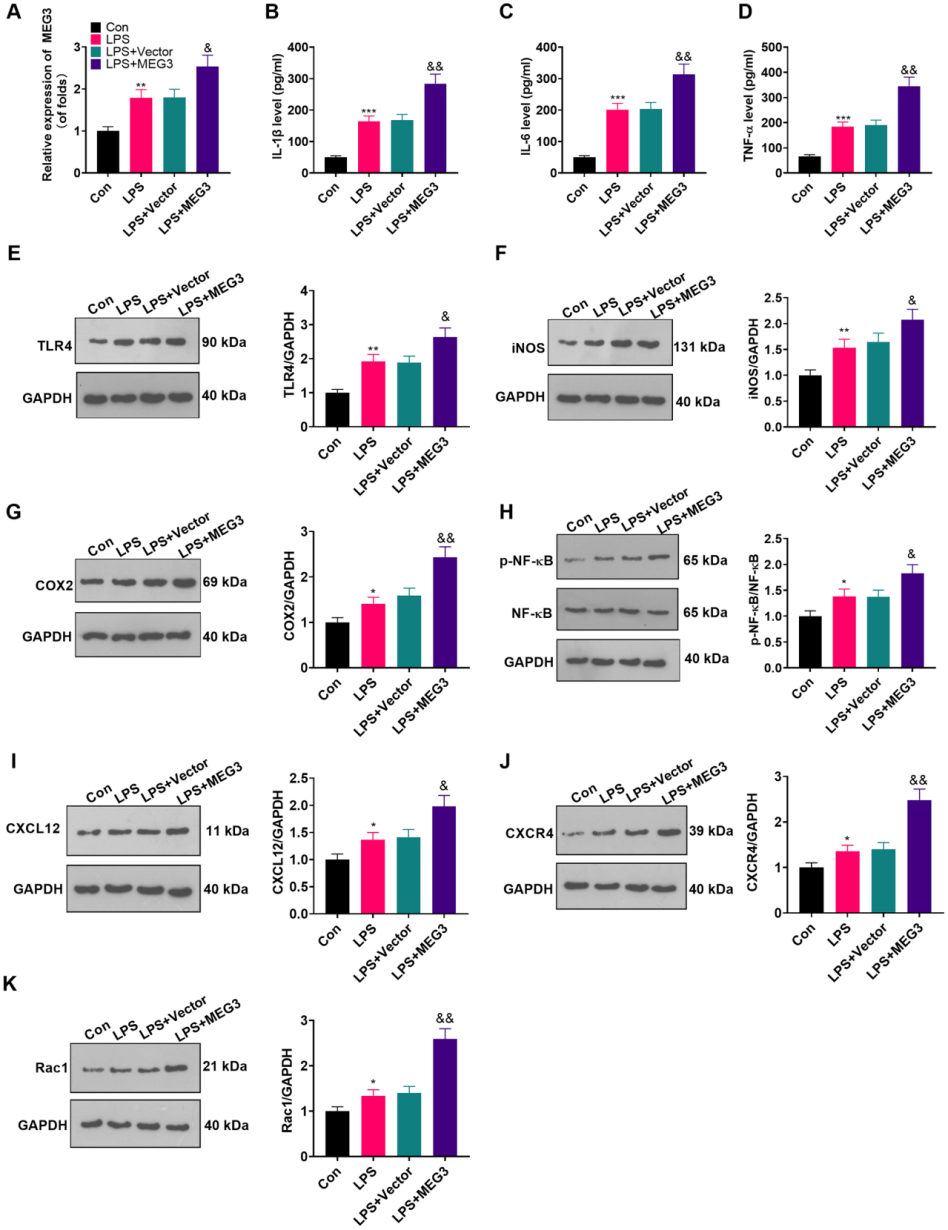
**Up-regulating MEG3 facilitated LPS-induced astrocyte inflammation.** (**A**) After transfection of MEG3 overexpressing plasmids, the level of MEG3 in astrocytes was tested by RT-qPCR. (**B**–**D**) The contents of IL-1β (**B**), IL-6 (**C**), TNF-α (**D**) in astrocytes were monitored by ELISA after MEG3 up-regulation. (**E**–**H**) The levels of TLR4 (**E**), iNOS (**F**), COX2 (**G**), NF-κB (**H**), CXCL12 (**I**), CXCR4 (**J**), and Rac1 (**K**) in astrocytes after up-regulation of MEG3 were compared by WB. Data were expressed as mean±SD. n=3. **P<0.01, ***P<0.001 (vs. LPS group). &*P*<0.05, &&*P*<0.01 (vs. LPS+Vector group).

To make certain the function of MEG3 in cells, we transfected MEG3 overexpression plasmids and control vectors (NC-vector) into LPS-induced astrocytes. RT-qPCR demonstrated that MEG3 was up-regulated after transfection with the MEG3 overexpression plasmids compared to that of the LPS+Vector group (Figure 3A; *P*<0.05). The expressions of inflammatory cytokines and inflammatory proteins in LPS-induced astrocytes was determined by ELISA and WB. The results uncovered that inflammatory factors (including IL-1β, TNF-α and IL-6) (Figure 3B–3D; *P*<0.05), as well as inflammatory proteins (including TLR4, iNOS, COX2, p-NF-κB, CXCL12, CXCR4, and Rac1) (Figure 3E–3K; *P*<0.05) were up-regulated in the LPS group (vs. the control group). Additionally, after transfection of MEG3 overexpression plasmids, these inflammatory factors and inflammatory proteins were further increased (Figure 3B–3K; *P*<0.05 vs.LPS+Vector group). Thus, up-regulating MEG3 increased the levels of inflammatory cytokines at the cellular level in astrocyte.

### Overexpressing miR-130a-5p impeded LPS-induced astrocyte inflammation

**Figure 4:**
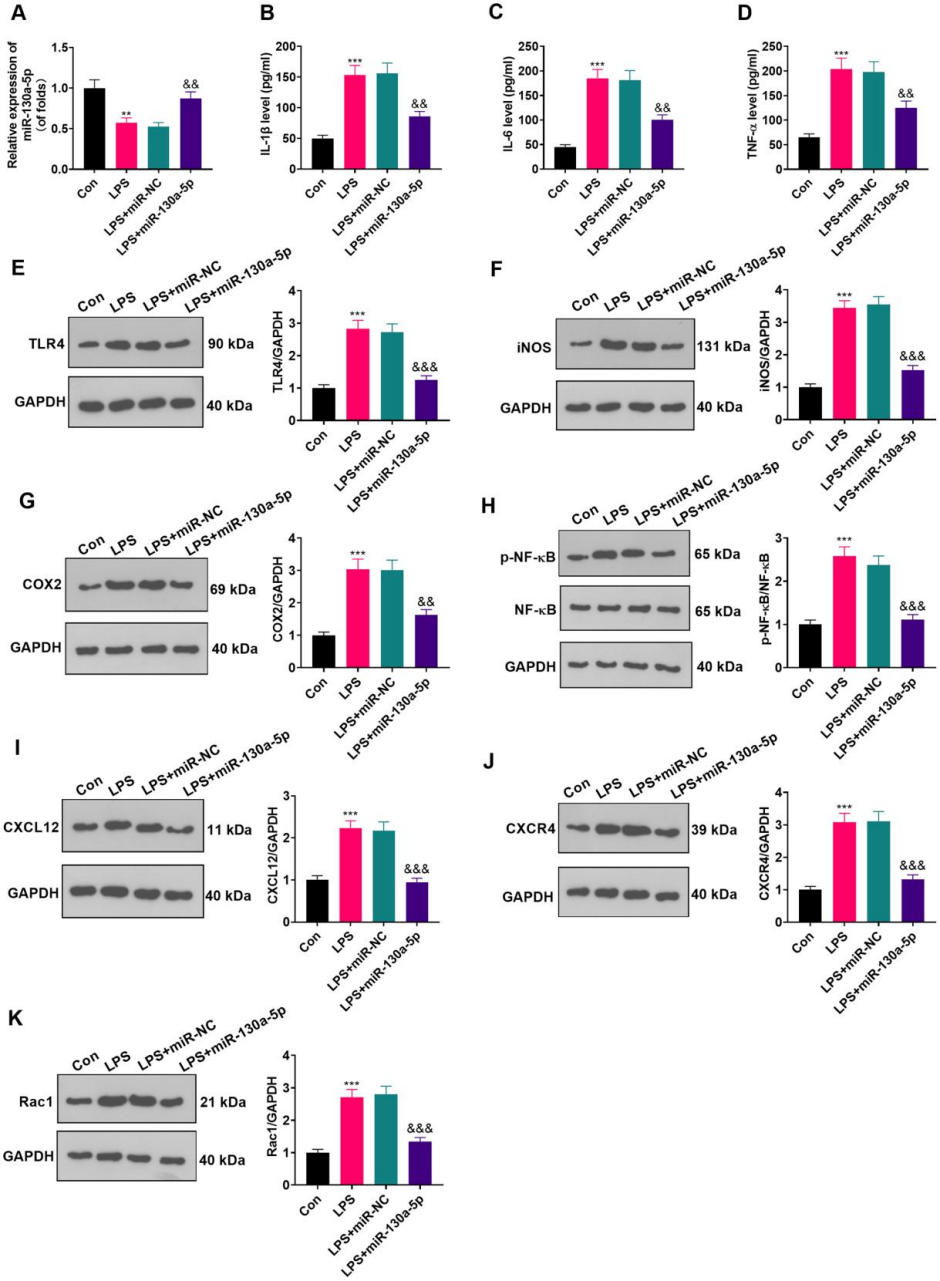
**Up-regulating miR-130a-5p suppressed LPS-induced inflammation in astrocytes.** (**A**) After transfection of miR-130a-5p mimics, the mRNA level of miR-130a-5p in astrocytes was tested by RT-qPCR. (**B**–**D**) The contents of IL-1β (**B**), IL-6 (**C**) and TNF-α (**D**) in astrocytes were compared by ELISA after up-regulation of miR-130a-5p. (**E**–**H**) WB detected the expression of TLR4 (**E**), iNOS (**F**), COX2(**G**), NF-κB (**H**), CXCL12 (**I**), CXCR4 (**J**), and Rac1 (**K**) in astrocytes after up-regulating miR-130a-5p. Data were expressed as mean±SD. n=3. **P<0.01, ***P<0.001 (vs. LPS group). &*P*<0.05, &&*P*<0.01 (vs. LPS+miR-NC group).

To clarify the role of miR-130a-5p in astrocytes, we transfected miR-130a-3p mimics and controls into LPS-induced astrocytes and checked the miR-130a-3p profile by RT-qPCR. The results illustrated that miR-130a-3p expression was heightened after miR-130a-3p mimics’ transfection compared with the LPS+miR-NC group, demonstrating successful transfection (Figure 4A; *P* < 0.05). Then, the biological role of miR-130a-5p in NP was examined by assessing the expression of IL-6, IL-1β and TNF-α. As displayed in Figure 4B–4D, up-regulating miR-130a-3p attenuated the levels of IL-6, IL-1β and TNF-α in astrocytes. Additionally, up-regulating miR-130a-3p distinctly hampered the levels of TLR4, iNOS, COX2, NF-κB phosphorylation, CXCL12, CXCR4, and Rac1 in astrocytes (Figure 4E–4K; *P*<0.05 vs.LPS+miR-NC group). These outcomes corroborated that overexpressing miR-130a-5p hampered inflammation in LPS-induced astrocytes.

### MEG3 targeted miR-130a-5p

**Figure 5:**
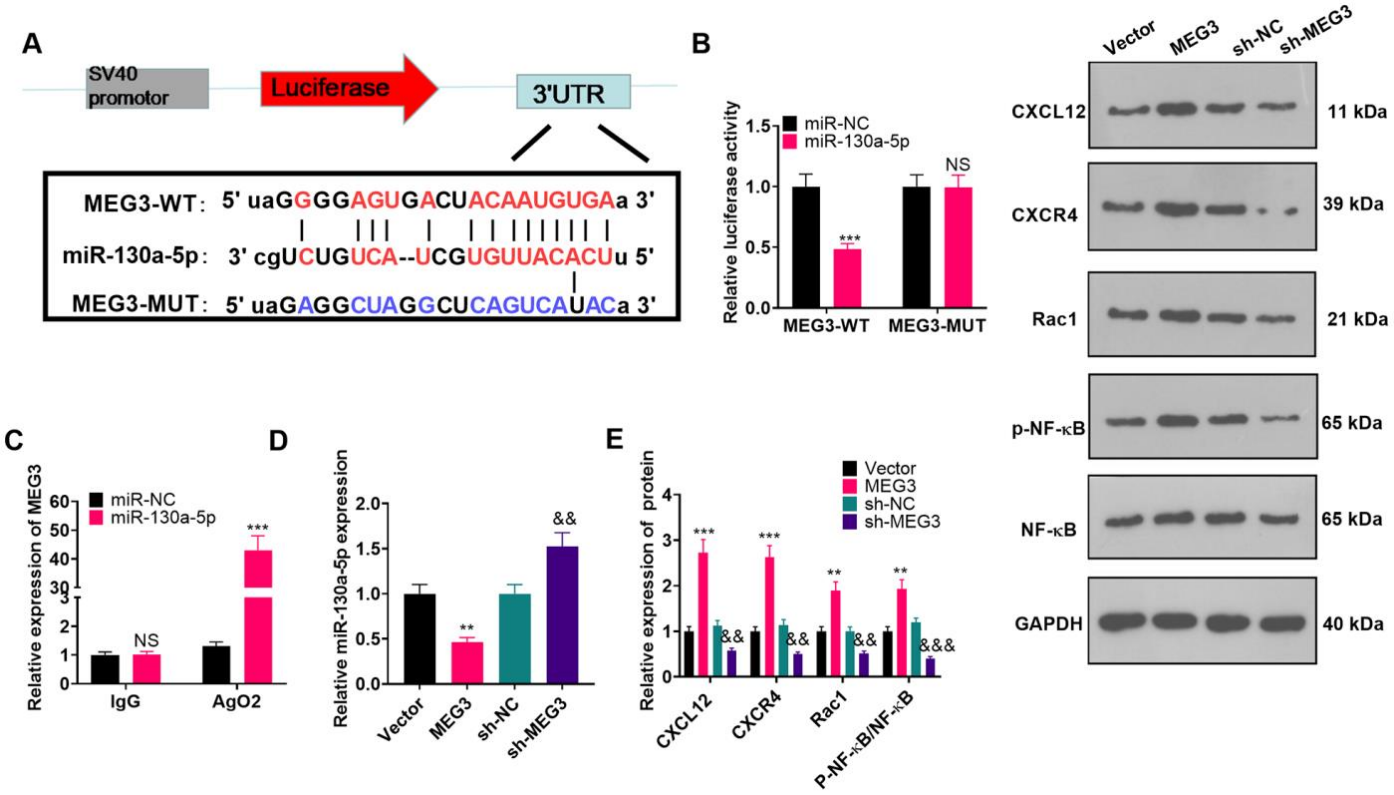
**MEG3 targeted miR-130-a-5p.** (**A**) Binding locations between MEG3 and miR-130a-5p were predicted using the starbase v2.0 database. (**B**) The target association between MEG3 and miR-130a-5p in astrocytes was clarified by the dual-luciferase reporter assay. (**C**) RIP was implemented to monitor the binding relationship between MEG3 and miR-130a-5p in astrocytes. NS*P*>0.05, ***P<0.001(vs. miR-NC group). (**D**) The miR-130a-5p expression in astrocytes after up-regulation or down-regulation of MEG3 was gauged by RT-qPCR. (**E**) WB was conducted to test the expression of CXCL12, CXCR4, Rac1 and NF-κB in astrocytes after up-regulating or down-regulating MEG3. Data were expressed as mean±SD. n=3. **P<0.01, ***P<0.001 (vs. Vector group). &*P*<0.05, &&*P*<0.01 (vs. sh-NC group).

To investigate the molecular mechanism of MEG3’s promoting NP in CCI rats, we predicted miRNAs through the ENCORI (http://starbase.sysu.edu.cn/) database and identified miR-130a-5p as a possible target of MEG3 (Figure 5A). The sequences of wt-MEG3 and mut-MEG3 were constructed into the luciferase reporter vectors. As exhibited, the fluorophore activity was declined in cells cotransfected with miR-130a-5p mimics and wt-MEG3, whereas no obvious effect was observed in the mut-MEG3 group. RIP revealed that miR-130a-5p and MEG3 were dramatically enriched by anti-Ago2 but not anti-IgG in astrocytes (Figure 5C; *P*< 0.05). To ascertain the relationship between miR-130a-5p and MEG3 in astrocytes, we examined the expression of miR-130a-5p as well as CXCL12/CXCR4 and its downstream Rac1 and NF-κB in astrocytes by RT-qPCR and WB after transfection with MEG3 overexpression plasmids or sh-MEG3. As a result, up-regulating MEG3 restrained the miR-130a-5p expression in astrocytes, while down-regulating MEG3 exerted the opposite effects (Figure 5D; *P*< 0.05). Meanwhile, MEG3 overexpression was positively related to the higher expression of CXCL12/CXCR4 and its downstream Rac1, and NF-κB phosphorylation. Contrarily, MEG3 knockdown attenuated CXCL12, CXCR4, Rac1, and NF-κB phosphorylation (Figure 5E; *P*<0.05 vs. sh-NC group). The above results confirmed that MEG3 targeted and inhibited miR-130a-5p.

### Overexpressing miR-130a-5p reversed the inflammatory response in MEG3-mediated astrocytes

**Figure 6:**
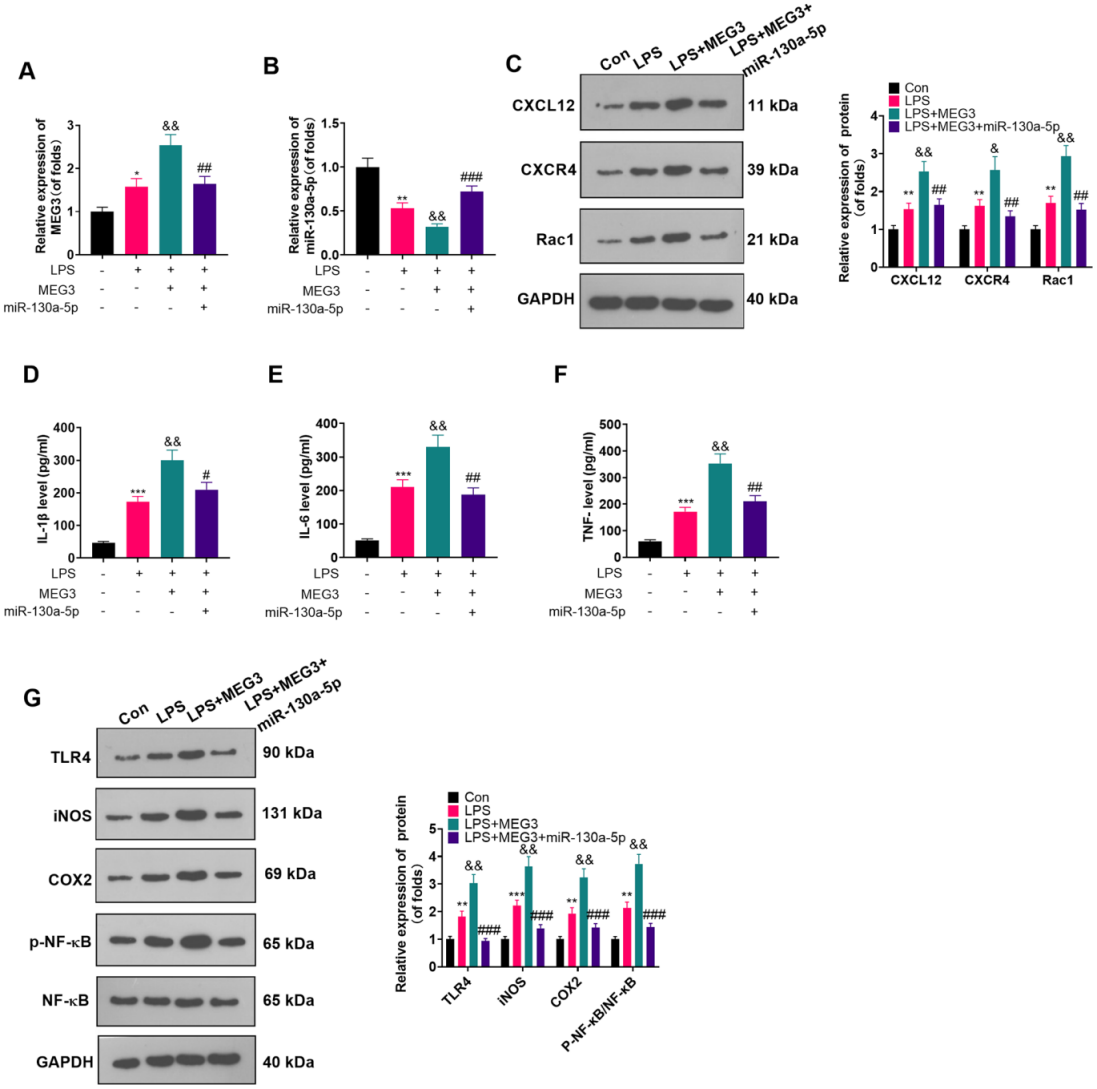
**Overexpressing miR-130a-5p reversed the MEG3-mediated astrocyte inflammation.** MEG3 overexpression plasmids and miR-130a-5p mimics were transfected in LPS-induced astrocytes. (**A**, **B**) The mRNA profiles of MEG3 and miR-130a-5p in astrocytes were tested by RT-qPCR. (**C**) The protein levels of CXCL12, CXCR4, and Rac1 in astrocytes were examined by WB. D-F: The concentrations of IL-1β (**D**), IL-6 (**E**) and TNF-α (**F**) in astrocytes were determined by ELISA. (**G**) WB was utilized to test the expression of TLR4, iNOS, COX2 and NF-κB in astrocytes. Data were expressed as mean±SD. n=3. *P<0.05, **P<0.01, ***P<0.001 (vs. Con group), #*P*<0.05, ##*P*<0.01 (vs. LPS group), &*P*<0.05, &&*P*<0.01, &&&*P*<0.001 (vs. LPS+MEG3 group).

The connection between miR-130a-5p and MEG3 in cells had been verified previously. To clarify whether MEG3 interacted with CXCL12 through miR-130a-5p, we added miR-130a-5p mimics in LPS-induced astrocytes and gauged the expression of MEG3, miR-130a-5p and CXCL12/CXCR4 and its downstream factors Rac1 and NF-κB by RT-qPCR and WB. As expected, compared with the LPS+MEG3 group, transfection of miR-130a-5p mimics resulted in up-regulation of miR-130a-5p and down-regulation of MEG3, CXCR4, and Rac1 (Figure 6A–6C; *P*<0.05). Besides, in comparison to the LPS+MEG3 group, the levels of inflammatory factors (Figure 6D–6F; *P*<0.05) and inflammatory proteins, including TLR4, iNOS, COX2, and phosphorylated NF-κB, were substantially attenuated after transfection with miR-130a-5p mimics (Figure 6G; *P*< 0.05 vs. the LPS+MEG3 group). These results manifested that MEG3 aggravated inflammation of LPS-induced astrocytes by targeting the miR-130a-5p/CXCL12 axis.

## DISCUSSION

NP originates from the pathological nervous system, clinically characterized by spontaneous pain (persistent, paroxysmal) and induced pain (hyperalgesia, abnormal pain) [29]. Reactive astrocyte-mediated neuroinflammation in the spinal dorsal horn contributes to NP [30]. Several studies have confirmed that inactivating astrocytes alleviates NP [31, 32]. LPS has been widely applied to activate glia *in vitro* [33]. Therefore, we employed CCI and LPS to construct experimental models in rats and astrocytes, respectively. This study revealed that MEG3 was up-regulated in spinal cord tissues of CCI rats and LPS-induced astrocytes. Furthermore, MEG3 activated the CXCL12/CXCR4 pathway by sponging miR-130a-5p, so as to aggravate NP in CCI rats and induce astrocytes. Our study may provide a new therapeutic target for NP.

Emerging evidence reveals that lncRNAs contribute to NP [34]. For instance, lncRNA XIST [35], lncRNA LINC00657 [36], and lncRNA NEAT1 [37] are all up-regulated in the CCI-induced NP model. Down-regulating them significantly lightens NP by repressing inflammatory response through attenuation of COX-2, TNF-α and IL-6. Additionally, lncRNAs play a prominent role in mediating astrocyte activation. Inhibition both lncRNA SNHG5 [38] and lncRNA GM14461 [39] dampens NP progression by inactivating astrocytes. On the other hand, MEG3 is widely involved in neuroinflammation. For example, up-regulating MEG3 ameliorates cognitive impairment, attenuates neuronal damage and abates astrocyte activation in Alzheimer's disease (AD) rat hippocampal tissues by choking the PI3K/Akt pathway [40]. Besides, MEG3 is up-regulated in traumatic brain injury (TBI), and up-regulated MEG3 boosts microglial activation and exacerbates TBI [12]. Meanwhile, MEG3 is highly expressed in the MACO rat model. Inhibiting the MEG3 expression improves the nerve injury and reduces the cerebral infarction area, blood-brain barrier permeability and neuronal apoptosis of MCAO rats [41]. Those studies confirmed that MEG3 has remarkable significance in affecting neuroinflammation. Nevertheless, the specific role of MEG3 in NP of CCI model rats remains unclear. Here, we discovered that MEG3 was up-regulated in spinal cord tissues of CCI rats and LPS-treated astrocytes. Through intrathecal injection of LV-MEG3 lentivirus, we ascertained that up-regulating MEG3 in the CCI model aggravated NP and elevated the levels of IL-1β, TNF-α and IL-6 in rats.

Dysregulation of miR-130a-5p is associated with diversified inflammatory diseases [42–44]. Additionally, miR-130a-3p is signally overexpressed in rats with spinal cord injury (SCI)-induced NP and in LPS-induced BV2 microglia. Inhibiting miR-130a-3p expression activates IGF-1/IGF-1R, thereby abating SCI-induced NP [45]. Nevertheless, our study yielded different results, which were related to different downstream targets of miR-130a-5p. We found that miR-130a-5p expression decreased time-dependently in spinal cord tissues of CCI rats and LPS-induced astrocytes [25]. In parallel, miR-130a-5p mimics’ transfection elevated miR-130a-5p expression, which suppressed LPS-induced inflammation in astrocytes.

CXCL12 is a specific chemokine ligand that contributes to cell chemotaxis. Upon binding to CXCR4, CXCL12 activates different signaling cascades to modulate cell proliferation, migration and metabolism [46]. The CXCL12/CXCR4 signal axis is involved in modulating NP [47]. Mai CL et al. found that CXCL12 gained an elevated level in circulating monocytes and plasma of patients with chronic pain. Blocking the CXCL12-CXCR4 signal transduction reduces the aggregation of peri-cerebrovascular macrophages in mouse hippocampus induced by nerve injury and alleviates neuroinflammation and resultant NP [48]. The expression of CXCL12 and CXCR4 in L4-5 spinal dorsal horn of SNI rats is up-regulated. Administration of CXCR4 antagonist AMD3100 declines the mechanical hypersensitivity induced by SNI rats [49], which has also been confirmed in previous studies [50, 51]. Here, CXCL12/CXCR4 is up-regulated in CCI rats, and overexpressing miR-130a-5p attenuates the MEG3-mediated inflammation in astrocytes by impeding CXCL12/CXCR4 expression.

The toll-like receptor 4 (TLR4)/nuclear factor-κB (NF-κB) axis is activated following CCI-induced NP [52]. It is often recognized as a vital pathway in charge of inflammatory cytokines expression and release [53]. Targeting the TLR4 pathway improves NP. For instance, intrathecal administration of LvOn-TLR4 small interfering (si)RNA (si-TLR4) prevents allodynia and hyperalgesia in CCI rats [54]. In another study, tizanidine, a highly selective α2-adrenoceptors (α2-AR) agonist, dampens spared nerve injury (SNI)-induced mechanical and thermal hyperalgesia by restraining the production of IL-1β, IL-6 and TNF-α, as well as the activation of TLR4/NF-κB [55]. Additionally, targeting CXCR4-mediated inflammation is associated with TLR4 signal inhibition [56, 57]. Our study indicated that MEG3 up-regulated the CXCL12/CXCR4/Rac1 axis and the TLR4/NF-κB pathway by abating miR-130a-5p. miR-130a-5p overexpression choked CXCL12/CXCR4/Rac1 and suppressed TLR4/NF-κB.

Overall, our study suggests that MEG3/miR-130a-5p/CXCL12/CXCR4 was altered in the CCI-induced NP rat model. Up-regulating MEG3 exacerbates NP and intensifies neuroinflammation. Moreover, MEG3 targets miR-130a-5p to elevate CXCL12/CXCR4 expression, which provides a potential prognostic marker and target for NP.
